# Risk of Aspiration Pneumonitis After Elective Esophagogastroduodenoscopy in Patients on Glucagon-Like Peptide-1 Receptor Agonists

**DOI:** 10.7759/cureus.66311

**Published:** 2024-08-06

**Authors:** Ruaa Al Sakka Amini, Abdel-Latif S Ismail, Maysarah Al-Aqrabawi, Wesam Aleyadeh, Abdul Mohammed, Nadera Altork, Hazem Abosheaishaa, Khaled A Elfert, Spencer R Goble, Bisher Sawaf, Saurabh Chandan

**Affiliations:** 1 Nephrology, Abdali Hospital, Amman, JOR; 2 Internal Medicine, University of Maryland Medical Center, Baltimore, USA; 3 Anesthesia and Pain Management, Sulaiman Al-Habib Medical Group, Riyadh, SAU; 4 Medicine, Cleveland Clinic Akron General, Akron, USA; 5 Gastroenterology and Hepatology, AdventHealth Orlando, Orlando, USA; 6 Medicine, Medstar Health/Georgetown-Washington Hospital Center, Washington, USA; 7 Internal Medicine, Icahn School of Medicine at Mount Sinai, Queens Hospital Center, New York, USA; 8 Internal Medicine/Gastroenterology, Cairo University, Cairo, EGY; 9 Internal Medicine, St. Barnabas Hospital Health System, New York, USA; 10 Internal Medicine, Hennepin Healthcare, Minneapolis, USA; 11 Internal Medicine, Hamad Medical Corporation, Doha, QAT; 12 Gastroenterology and Hepatology, Creighton University, Omaha, USA

**Keywords:** aspiration pneumonitis, glp-1ra, endoscopy, egd, esophagogastroduodenoscopy

## Abstract

Background

Glucagon-like peptide-1 receptor agonists (GLP-1RAs) are gaining popularity in the management of diabetes mellitus and obesity. It has been suggested that this class of medications causes delayed gastric emptying which raised concerns about the potential for aspiration of gastric contents in patients undergoing sedation. This led to a statement by the American Society of Anesthesiologists about their preoperative use. Nevertheless, there is minimal evidence regarding the effects of GLP-1RAs on the risk of aspiration post-esophagogastroduodenoscopy (EGD). In this study, we sought to evaluate the incidence of aspiration and pneumonia in patients receiving GLP-1RAs who underwent EGD.

Methodology

We performed a retrospective cohort study in TriNetX, a global federated research network of electronic health records. The primary outcome was the development of aspiration post-EGD. Secondary outcomes were the development of aspiration pneumonia and requiring antibiotics post-EGD. One-to-one propensity score matching was performed for age, sex, diabetes mellitus, obesity, and other comorbidities between the cohorts.

Results

Our analysis showed a small but significant risk of aspiration pneumonitis in patients on GLP-1RAs undergoing elective EGD compared to non-GLP-1RA-receiving patients. However, there was no increased risk of the composite outcome of respiratory failure or intensive care unit (ICU) admission; however, this did not reach statistical significance.

Conclusions

GLP-1RA use was associated with an increased risk of aspiration in patients undergoing elective upper endoscopy. However, this did not translate to an increased risk of respiratory failure or ICU admission. Our findings highlight the importance of following an individualized approach to preoperative management that takes into consideration GLP-1RA indications and other aspiration risk factors, including advanced age, impaired gag reflex, and gastrointestinal symptoms such as nausea and abdominal distention.

## Introduction

Glucagon-like peptide-1 receptor agonists (GLP-1RAs) have been gaining popularity in managing diabetes mellitus (DM) owing to their effectiveness, cardioprotection, and convenient dosing [1]. Moreover, GLP-1RAs have consistently shown promising outcomes in the reduction of body weight in obese individuals with and without diabetes [2]. Yet, despite their widely recognized effects on weight loss, the mechanism underlying these effects is not entirely understood but seems to be neurally mediated. It has been demonstrated that GLP-1RA interferes with the central parasympathetic outflow, hindering gastropancreatic function.

Their known mechanisms of action include slowing gastric transit, promoting hyperglycemia-induced insulin secretion, and suppressing glucagon secretion in hyperglycemia [1]. Slowing gastric transit results in gastrointestinal (GI) symptoms, including abdominal pain, bloating, vomiting, pancreatitis, intestinal obstruction, and a higher risk of gastroparesis [3]. Additionally, slower gastric transit and delayed gastric emptying are thought to predispose to aspiration of gastric contents, especially in patients undergoing sedation [1]. Two case reports have shown incidents of aspiration under anesthesia in a 42-year-old male and a 48-year-old female who were taking semaglutide injections for weight loss before undergoing upper GI endoscopy and breast surgery, respectively, despite a long preoperative fasting period of 20 hours [4,5].

A consensus-based guidance by the American Society of Anesthesiologists (ASA) on the preoperative management of patients on GLP-1RA suggested holding the medication dose on the day of the procedure in patients on daily dosing and holding the dose one week before the procedure in patients on weekly dosing [6].

However, clinical trials have shown a low rate of aspiration during upper GI endoscopies in individuals taking GLP-1RAs [7], and GI societies suggest personalized approaches when contemplating endoscopy for this group of patients [8].

Robust evidence of the effect of GLP1-RAs on the risk of aspiration in patients undergoing sedation is lacking. In this study, we aim to evaluate the risk of aspiration pneumonitis in patients receiving GLP-1RAs who underwent elective esophagogastroduodenoscopy (EGD).

## Materials and methods

Design and data source

The multi-institutional database TriNetX (Cambridge, MA, US) was used for this retrospective cohort study. TriNetX is a worldwide federated research network that offers real-time access to electronic health records (EHRs) of roughly 213 million patients across 92 healthcare organizations in the United States [9]. Clinical variables are extracted from clinical documents using an integrated natural language processing system and EHRs. To protect patient health information, the interface only offers statistical summaries and aggregate counts, guaranteeing that the data is de-identified throughout.

Study population

A real-time search of the Diamond Network in the TriNetX platform was conducted from June 2013 until June 2023. Patient cohorts included adult patients (age ≥18 years) who underwent outpatient EGD. The outpatient settings were ensured by excluding patients who had an inpatient Current Procedural Terminology (CPT) code in the preceding week. We used the following CPT codes: 1013659, 1013699, and 1013729. Patients who underwent EGD were identified using the following CPT codes for EGD: 1021431 and 43235. We identified the GLP-1RA cohort using the following RxNorm codes: dulaglutide: 1551291, liraglutide: 475968, semaglutide: 1991302, exenatide: 60548, lixisenatide: 1440051, and albiglutide: 1534763. Patients must have had GLP-1RAs started before the index EGD to be included in the study. TriNetX allows establishing temporality between different groups within a cohort, which enabled us to determine which patients were taking GLP-1RAs before undergoing the index EGD. Importantly, patients with a history of gastrostomy, gastroparesis, altered esophagus or stomach anatomy, dysphagia, achalasia, opioid use, and cerebral palsy were excluded from the study.

Outcomes

The study’s primary objective was to assess the risk of aspiration pneumonitis following outpatient EGD in patients taking GLP-1RAs. The risk of aspiration pneumonitis was evaluated within the first 24 and 48 hours post-EGD. Aspiration pneumonitis diagnosis was identified using the International Classification of Disease, Tenth Revision, and Clinical Modification (ICD-10-CM) code J69.0. The secondary outcome was the composite outcome of developing respiratory failure, ICD-10-CM code: J96, or requiring critical care services, CPT codes: 1013729, 1014309, and 99291.

Statistical analysis

All statistical analyses were conducted using the TriNetX software using the browser-based, real-time analytics feature, TriNetx Live (TriNetX LLC, Cambridge, MA) [9]. Baseline characteristics of cohorts were described using means, standard deviations, and proportions. Covariates based on demographics, comorbid diseases, and medications were identified. One-to-one (1:1) propensity score matching (PSM) was performed to balance the following covariates between groups: age, sex, obesity, DM, and nicotine dependence.

These covariates were chosen because they were significantly different between the two groups. TriNetX platform uses input matrices of the user-identified covariates to conduct logistic regression analysis to obtain propensity scores for all subjects [9]. The generated propensity scores were utilized for patient matching through greedy nearest-neighbor algorithms, employing a caliper width equivalent to 0.1 pooled standard deviation. TriNetX shuffles the row order to mitigate bias from nearest-neighbor algorithms. Following the PSM, the risk for each outcome was computed and presented as adjusted odds ratios (aORs) alongside 95% confidence intervals (CIs). Two-sided p-values of less than 0.05 were considered statistically significant.

## Results

Patient characteristics

We identified 59,323 patients in the GLP-1RA cohort and 5,890,919 patients in the control cohort. The GLP-1RA cohort had a higher mean age (59.9 vs. 54.6 years, p < 0.0001) and a lower proportion of female sex (45.3% vs. 47.8%, p < 0.0001) compared to the control cohort. After PSM, there were 59,323 patients in each cohort (Table 1).

**Table 1:** A comparative analysis of covariates of patients who underwent elective EGD between those who were on GLP-1RAs and those who were not, before and after propensity matching. After propensity score matching, the risk of each outcome was calculated and expressed as adjusted odds ratios with 95% confidence intervals. Two-sided p-values <0.05 were considered statistically significant. EGD: esophagogastroduodenoscopy; DM: diabetes mellitus; GLP-1RA: glucagon-like peptide-1 receptor agonist

Variable	GLP-1RA	No GLP-1RA	P-value	Standard difference	GLP-1RA	No GLP-1RA	P-value	Standard difference
	Before propensity matching				After propensity matching			
Number of patients	59,323	5,890,919	-	-	59,323	59,323	-	-
Age at index EGD (years)	59.9 ± 11.4	54.6 ± 17.2	<0.001	0.3624	59.9 ± 11.4	59.9 ± 11.4	1.000	<0.0001
Female sex (%)	26,898 (45.3%)	2,820,834 (47.8%)	<0.0001	0.01510	26,898 (45.3%)	26,898 (45.3%)	1.000	<0.0001
DM (%)	51,535 (86.8%)	1,059,011 (17.9%)	<0.0001	1.9053	51,535 (86.8%)	51,535 (86.8%)	1.000	<0.0001
Obesity/Overweight (%)	30,705 (51.7%)	975,770 (16.5%)	<0.0001	0.7992	30,705 (51.7%)	30,705 (51.7%)	1.000	<0.0001
Nicotine dependence (%)	12,202 (20.5%)	564,253 (9.5%)	<0.0001	0.3109	12,202 (20.5%)	12,202 (20.5%)	1.000	<0.0001

Risk of aspiration post-esophagogastroduodenoscopy

Our analysis showed a small risk of aspiration pneumonitis in patients on GLP-1RA undergoing elective EGD; however, it was significantly increased compared to the control group. After PSM, 10 (0.017%) patients in the GLP-1RA group developed aspiration pneumonitis within 24 hours of the EGD compared to zero patients in the control group (Table 2). Patients who had the outcome before the time window were excluded from the results. Similar results were obtained when the analysis was run to include outcomes within 48 hours post-EGD.

**Table 2:** Clinical outcomes for propensity-matched GLP-1RA and non-GLP-1RA cohorts who underwent elective EGD in the United States within 24 hours post-EGD. After propensity score matching, the risk of each outcome was calculated and expressed as adjusted odds ratios with 95% confidence intervals. Two-sided p-values <0.05 were considered statistically significant. ICU: intensive care unit; GLP-1RA: glucagon-like peptide-1 receptor agonist; EGD: esophagogastroduodenoscopy; N/A: not available

Outcome	Cohort	Total number of patients	Proportion of patients (%)	Adjusted odds ratio	95% confidence interval	P-value
Aspiration	GLP-1RA	10	0.017%	N/A	N/A	N/A
	Control	0	0	-	-	-
Respiratory failure or ICU admission	GLP-1RA	16	0.029%	0.75	0.396-1.455	0.4061
	Control	21	0.038%	-	-	-

Risk of respiratory failure or intensive care unit admission post-esophagogastroduodenoscopy

On the other hand, our analysis showed no increased risk of the composite outcome of respiratory failure or intensive care unit (ICU) admission within 24 or 48 hours post-EGD in patients on GLP-1RAs. After PSM, 16 (0.029%) patients from the GLP-1RA cohort had the composite outcome of respiratory failure or ICU admission within 24 hours post-EGD compared to 21 (0.038%) patients from the control group (aOR = 0.75, 95% CI = 0.396-1.455, p = 0.4061; however, these results were not statistically significant. Patients who had the outcome prior to the time window were excluded from the results. Similar results were obtained when the analysis was run to include outcomes within 48 hours post-EGD.

## Discussion

GLP-1RAs have emerged as prominent therapeutic agents in diabetes management, offering significant benefits in weight loss and improved cardiovascular health [10]. There are currently seven licensed GLP1-RAs, which have different mechanisms of action depending on their duration of action and whether they are short-acting or long-acting. Exenatide and lixisenatide are two examples of short-acting medications that decrease postprandial hyperglycemia mainly by slowing gastric emptying. Long-acting medications such as liraglutide, semaglutide, dulaglutide, and exenatide long-acting release, comparatively, boost insulin secretion and decrease glucagon to control postprandial hyperglycemia [1].

Clinically, however, a recent study indicated that individuals using long-acting GLP1-RAs showed a notably greater amount of gastric residue compared to those not receiving GLP1-RA treatment, as observed through EGD in diabetic patients. This suggests that the impact of long-acting GLP1-RAs on gastric emptying could be significant [11].

However, this effect can vary depending on the specific analog used. For instance, medications such as liraglutide and semaglutide are associated with a more pronounced delay in gastric emptying compared to exenatide. The average gastric emptying time can extend by 30-60 minutes in patients on GLP-1RAs compared to those not on these medications. This variability among GLP-1RAs might influence the risk of aspiration, as different agents have different impacts on GI motility [3].

More recently, semaglutide has been studied to determine if its effect on gastric emptying occurs in a dose-dependent manner, particularly at high dosages (≥1.0 mg) [12].

Sodhi et al. conducted a recent study to examine the GI side effects linked to the use of GLP-1 RAs for non-diabetic purposes, particularly focusing on biliary disease, pancreatitis, intestinal obstruction, and gastroparesis. Using data from the PharMetrics Plus database covering the years 2006 to 2020 and involving a random sample of 16 million patients, the study compared individuals using the GLP-1RAs semaglutide and liraglutide with individuals using bupropion-naltrexone, a weight loss medication not related to GLP-1RAs. It is important to note that this study did not include diabetic patients. The research revealed a higher risk of pancreatitis, bowel obstruction, and gastroparesis among GLP-1RA users, though there was no significant increase in the risk of biliary disease [3].

Despite the widespread use of GLP-1RAs, their evident side effect of delayed gastric emptying may increase the aspiration risk of gastric contents, especially during procedures that require sedation [1,6,10]. We found a small but comparatively increased aspiration pneumonitis risk in the GLP-1RA cohort up to 48 hours following an elective EGD. Our findings support the current consensus [6] regarding the timely cessation of these drugs before the procedure.

Traditionally, the risk of aspiration pneumonitis following elective EGD has been considered low. Bohman et al. [13] reported a risk of pulmonary aspiration in patients undergoing elective EGD of 4.6 per 10,000. They concluded that aspiration events were associated with increased mortality, risk of ICU admission, and longer hospital stays. A similar aspiration rate was reported in a retrospective study by Anazco et al. [7] conducted to assess the risk of aspiration pneumonitis following elective EGD in patients on GLP-1RAs. They reported that the outcome of pulmonary aspiration occurred in two out of 4,134 patients, with a rate of 4.8 cases per 10,000 endoscopies. Despite its uncommon occurrence, aspiration is a significant adverse event and can result in life-threatening complications such as pneumonia and respiratory failure [13,14].

The potential aspiration risk during EGD, the overall benefits of GLP-1RA, and the associated risks with holding them in patients with diabetes highlight a complex decision-making choice for providers. As the therapeutic landscape of GLP-1RA continues to evolve, further guidance from medical and anesthesiology societies is essential for managing patients undergoing sedation. The American Gastroenterology Association provided a rapid clinical practice update [8] in response to the ASA’s statement suggesting an individualized approach to preoperative management of GLP-1RA that takes into consideration their indication, patient’s GI symptoms, and other comorbidities and medications. The guidance statement suggested that patients on GLP-1RA who have followed standard perioperative procedures (typically an eight-hour solid-food fast and a two-hour liquid fast) and who do not have symptoms of nausea, vomiting, dyspepsia, or abdominal distention may proceed with upper and/or lower endoscopy.

Our findings stand out for having the largest patient population compared to existing research, incorporating multiple centers across the United States. Notably, the analysis comprehensively addressed various variables linked to aspiration, either through PSM, which allowed us to achieve a balanced distribution of covariates, or by utilizing robust exclusion criteria.

However, our study has multiple limitations. First, data sourced from TriNetX and EHRs are susceptible to entry errors, as well as the possibility of missing or underreported diagnoses, such as aspiration. It is essential to note that TriNetX relies on Rxnorm for medication data, which exclusively covers the US market. Moreover, dealing with procedures posed a distinct challenge due to the absence of a universally adopted standard for representing procedures, unlike the case with diagnoses.

Second, besides the retrospective design, which inherently limits the ability to establish causality between GLP-1RA use and aspiration risk, our study did not account for the pharmacokinetic and pharmacodynamic variations among different GLP-1RAs, which may influence the risk of delayed gastric emptying and aspiration. Future studies should stratify patients based on specific GLP-1RAs to better understand these differences.

Lastly, the low incidence of aspiration pneumonitis and respiratory failure events limits the statistical power to detect differences and highlights the need for larger studies or meta-analyses to achieve more precise estimates and confirm our findings.

## Conclusions

We found that GLP-1RA use was associated with an increased risk of aspiration in patients undergoing elective upper endoscopy. However, this did not translate to an increased risk of respiratory failure or ICU admission. Our findings highlight the importance of following an individualized approach to preoperative management that takes into consideration GLP-1RA indications and other aspiration risk factors, including advanced age, impaired gag reflex, and GI symptoms such as nausea and abdominal distention. Future prospective studies with detailed pharmacokinetic and pharmacodynamic data are necessary to fully understand the nuances of each GLP-1RA and their impact on gastric emptying and aspiration risk.
